# Artificial Intelligence: Readiness, Attitudes, and AI-Related Anxiety Among Oncology Nurses

**DOI:** 10.3390/healthcare14070848

**Published:** 2026-03-27

**Authors:** Elif Dönmez, Gamze Temiz, Burak Mete, Elif Marangoz, Tülay Ortabağ

**Affiliations:** 1 Oncology Nursing Department, Hamidiye Faculty of Nursing, University of Health Sciences, Istanbul 34668, Turkey; gamze.temiz@sbu.edu.tr; 2 Department of Public Health, Faculty of Medicine, Cukurova University, Adana 01330, Turkey; 3 Graduate Education Institute, Istanbul University-Cerrahpasa, Istanbul 34320, Turkey; elif.arslan5@saglik.gov.tr; 4 Faculty of Health Sciences, Istanbul Topkapi University, Istanbul 34662, Turkey; tulayortabag@topkapi.edu.tr

**Keywords:** artificial intelligence, nursing, anxiety, attitudes

## Abstract

**Objectives:** As artificial intelligence (AI) technologies become increasingly integrated into healthcare systems, understanding healthcare professionals’ psychological responses—particularly AI-related anxiety—has become increasingly important for the safe and effective implementation of these technologies in clinical practice. This study aimed to examine the relationships between oncology nurses’ readiness for artificial intelligence, their attitudes toward artificial intelligence, and their levels of AI-related anxiety. **Design:** A descriptive, cross-sectional study. **Setting:** An oncology hospital within a state hospital in Istanbul, Turkey. **Participants:** A total of 207 oncology nurses working full-time in clinical settings. **Methods:** Data were collected using an online survey consisting of a demographic information form, the Medical Artificial Intelligence Readiness Scale (MAIRS-MS), the Artificial Intelligence Anxiety Scale (AIAS), and the General Attitudes toward Artificial Intelligence Scale (GAAIS). Spearman correlation analysis, general linear modeling, and conditional mediation analysis were performed using JAMOVI (v2.6.17). A *p*-value of <0.05 was considered statistically significant. **Results:** AI-related anxiety was significantly and negatively correlated with both readiness and attitudes toward AI. General linear modeling showed that attitudes toward AI significantly predicted anxiety (β = −0.327, *p* < 0.001), whereas readiness did not have a direct significant effect. Conditional mediation analysis demonstrated that attitudes fully mediated the relationship between readiness and AI anxiety. The indirect effect of readiness on anxiety through attitudes was stronger among nurses who had received prior AI-related education. While the indirect effect remained significant among untrained nurses, its magnitude was considerably smaller. The total effect of readiness on anxiety was significant only in the untrained group, suggesting that structured education redirects the impact of readiness primarily through attitudes. **Conclusions:** Attitudes toward artificial intelligence represent the key psychological mechanism linking readiness to AI-related anxiety among oncology nurses. Prior AI education appears to strengthen this relationship by enhancing the association between readiness and attitudes and by being associated with lower anxiety levels. Educational and implementation strategies that emphasize ethical awareness and the development of positive, informed attitudes—rather than focusing solely on technical competence—are likely to be more effective in reducing anxiety and promoting the safe and ethical integration of AI into oncology nursing practice.

## 1. Introduction

Artificial intelligence (AI) is an umbrella term that encompasses a range of techniques developed to enable computers to emulate human-like cognitive functions, including learning, reasoning, communication, and decision-making [1]. In recent years, AI has emerged as a transformative and innovative tool in healthcare and has been increasingly adopted by healthcare professionals across multiple domains such as early diagnosis, treatment planning, clinical decision-making, education, and research [1,2,3]. Cancer remains a major public health concern worldwide due to its persistently high rates of mortality and morbidity [4]. Within this context, AI technologies offer significant promise as effective tools across the full spectrum of oncology nursing, including cancer prevention, early detection, treatment processes, survivorship, and end-of-life care [3,5].

As both primary users of AI-based technologies and providers of professional care, nurses play a pivotal role in shaping the development, implementation, and effective utilization of these technologies. Therefore, it is essential for nurses to understand how AI is applied in patient care, to possess adequate knowledge about these systems, and to embrace their integration into the care process [6]. However, the rapid integration of AI technologies presents not only new opportunities but also significant ethical, legal, and social responsibilities for the nursing profession [7]. Despite the potential benefits of artificial intelligence in healthcare, the rapid integration of these technologies may also generate uncertainty and concern among healthcare professionals. AI-related anxiety may arise from limited familiarity with AI systems, concerns about job displacement, and uncertainty regarding how these technologies may influence professional roles and clinical decision-making. Previous studies have reported that insufficient knowledge about AI technologies, uncertainty regarding their outcomes, and concerns about workforce implications may contribute to increased AI-related anxiety among healthcare professionals [8,9].

Nurses constitute the largest segment of the healthcare workforce, routinely document vast amounts of patient data, and are among the primary users of AI technologies. Nursing professional organizations have acknowledged the increasingly intertwined nature of nursing and technology and strongly emphasize the growing importance of AI in nursing practice [5,10]. While these organizations highlight the need to enhance nurses’ knowledge and competencies related to AI, the American Nurses Association (ANA), in its official position statement, underscores that nurses are responsible for ensuring that advanced technologies do not compromise the fundamental nature of human relationships in care delivery [10]. Furthermore, it is emphasized that nurses should be actively represented in the development and implementation of technologies, evaluate their impact on the quality of care, and anticipate the effects of AI on healthcare services. Oncology nurses, in their daily practice, routinely utilize a wide range of technologies, including electronic health records, remote monitoring systems, telehealth platforms, and mobile health applications. In addition, they support patients in using technologies such as online health services, social media, mobile applications, and wearable devices to enhance self-management [11]. When integrated into these digital tools, AI further enhances the effectiveness of cancer care and service delivery [11]. Moreover, AI applications hold significant potential for alleviating nurses’ heavy workloads and improving operational efficiency within clinical settings [5].

Artificial intelligence (AI) tools are generally accepted as technologies that will facilitate and enhance human work rather than replace physicians and other healthcare professionals, including nurses and midwives [12]. However, concerns that AI may become uncontrollable have given rise to a phenomenon known as AI anxiety. Fears related to potential job displacement, the belief that AI systems may operate autonomously, and perceptions of robots as threatening or fear-inducing entities may all contribute to the development of AI-related anxiety [13,14]. Such concerns may lead oncology nurses to develop prejudices against AI applications and potentially refrain from using technologies that could otherwise improve the quality of patient care. AI is widely emphasized as a force that can increase efficiency, create new opportunities, reduce human error, assume responsibility for solving complex problems, and perform repetitive and tedious tasks. Accordingly, these benefits of AI may create free time for individuals to learn, experiment, and explore, thereby enhancing human creativity and overall quality of life. For this reason, individuals are expected to demonstrate positive attitudes toward AI applications in these domains [14]. Following these discussions, the concept of attitude toward AI has emerged as a critical construct. In particular, individuals with higher levels of education and greater internet use have been shown to exhibit more positive attitudes toward AI [14,15]. In this context, “readiness for artificial intelligence in medicine” has been proposed as an indicator of the extent to which healthcare providers are prepared— in terms of knowledge, skills, and attitudes—to utilize AI applications alongside their professional competencies while delivering prevention, diagnosis, treatment, and rehabilitation services [16]. Nurses occupy a pivotal position in shaping and guiding the evolution of modern AI in healthcare delivery. Therefore, understanding nurses’ attitudes toward the use of these technologies is of critical importance [11].

The relationships between readiness, attitudes, and anxiety toward artificial intelligence can also be interpreted within the framework of the Technology Acceptance Model (TAM). According to TAM, individuals’ perceptions of a technology’s usefulness and ease of use shape their attitudes toward that technology, which subsequently influence their acceptance and behavioral responses [17]. In healthcare contexts, greater knowledge and readiness regarding emerging technologies such as artificial intelligence may foster more positive attitudes, which in turn can reduce uncertainty and technology-related anxiety among healthcare professionals. In this context, although several demographic determinants influencing attitudes toward AI have been documented in prior research, further studies are needed to better understand how these attitudes are shaped across different cultural settings. Although previous studies have examined artificial intelligence literacy, attitudes, or anxiety among healthcare professionals, limited research has simultaneously investigated the relationships between AI readiness, attitudes toward AI, and AI-related anxiety among practicing oncology nurses. Therefore, further research is needed to better understand how these factors interact in clinical nursing settings. Aimed at addressing this gap in the literature, the present study seeks to determine the effects of oncology nurses’ readiness for AI and their attitudes toward AI on their levels of AI-related anxiety. Gaining a deeper understanding of the impact of AI on nursing practice and establishing a robust foundation in this field will serve as a guiding framework for future research and clinical applications.

The research questions of the study are as follows:

- How is oncology nurses’ readiness for artificial intelligence associated with their levels of AI-related anxiety?
- How are oncology nurses’ attitudes toward artificial intelligence associated with their levels of AI-related anxiety?
- Do attitudes toward artificial intelligence mediate the relationship between oncology nurses’ AI readiness and AI-related anxiety?
- Does prior AI-related education moderate the relationship between AI readiness, attitudes toward AI, and AI-related anxiety?

## 2. Methods

### 2.1. Study Design, Sample and Setting

This study employed a descriptive and cross-sectional design and was conducted between February 2025 and July 2025 in an oncology hospital located within a state hospital in Istanbul. The oncology hospital has a capacity of 403 beds and employs a total of 432 oncology nurses. The study sample consisted of 207 nurses who met the inclusion criteria.

Eligible participants were oncology nurses working full-time at the hospital. Data were collected using a self-administered online survey distributed via Google Forms. The first page of the electronic survey included an information sheet describing the purpose of the study, assuring confidentiality, and emphasizing voluntary participation. The information sheet also clearly stated that refusal to participate would not affect employment status.

Participants were recruited using convenience sampling. Information about the study and the survey link was shared through the WhatsApp group of nurses working in the oncology hospital. The Google Forms link was distributed directly to the group, allowing only nurses working in the institution to access the survey. Throughout the data collection period, weekly reminder messages were posted in the WhatsApp group to improve participation. Nurses who were willing to participate accessed the survey voluntarily.

The target population of the study consisted of 432 oncology nurses working in the oncology hospital. The required sample size was calculated using an online sample size calculator (Raosoft Inc., Seattle, WA, USA). Assuming a 95% confidence level (Z = 1.96), a 5% margin of error (d = 0.05), and a conservative estimated proportion of 0.50 (*p* = 0.5), the minimum required sample size was determined to be 204 nurses. In the present study, data were obtained from 207 oncology nurses, which exceeded the calculated minimum and was therefore considered sufficient to represent the target population. This study was reported in accordance with the Strengthening the Reporting of Observational Studies in Epidemiology (STROBE) guidelines.

Ethical approval for this study was obtained from the University of Health Sciences Ethics Committee (No: 25/99). All procedures complied with the Declaration of Helsinki, ICH Guidelines for Good Clinical Practice, and international standards for human research protection.

### 2.2. Measurements

#### 2.2.1. Demographic Survey and Workplace Information

A questionnaire consisting of 21 items was developed by the researchers to assess participants’ sociodemographic characteristics and their views regarding artificial intelligence. The questionnaire included variables such as age, gender, educational level, and professional experience, as well as items related to the use of AI in daily life, trust in AI, and perceptions of AI as a potential professional threat.

#### 2.2.2. The Medical Artificial Intelligence Readiness Scale for Medical Students (MAIRS-MS)

Artificial intelligence readiness was assessed using the Medical Artificial Intelligence Readiness Scale, a psychometrically validated instrument developed by Karaca, Çalışkan, and Demir in 2021 to measure healthcare learners’ preparedness for AI technologies [16]. The scale consists of 21 items rated on a Likert-type format and incorporates four subdimensions—cognition (α = 0.830), ability (α = 0.770), vision (α = 0.723), and ethics (α = 0.632)—which together evaluate individuals’ conceptual understanding of AI, their capacity to effectively use AI-based tools in clinical practice, their perceptions regarding the opportunities and limitations of AI, and their adherence to ethical and legal standards relevant to AI use in healthcare. Higher scores reflect greater readiness for AI integration. The instrument has demonstrated strong psychometric properties, with an overall Cronbach’s alpha of 0.877 reported in the original validation study. In the present study, the scale was used to quantitatively assess oncology nurses’ readiness for artificial intelligence and to examine how readiness and attitudes toward AI relate to AI-related anxiety. Although the MAIRS-MS scale was originally developed for medical students, it assesses general competencies related to artificial intelligence—such as cognition, ability, vision, and ethics—that are relevant to a wide range of healthcare professionals. Therefore, the scale was considered appropriate for evaluating oncology nurses’ readiness for artificial intelligence in the present study. In the present study, the internal consistency of the scale was also examined to confirm its reliability in this sample.

#### 2.2.3. Artificial Intelligence Anxiety Scale (AIAS)

Artificial intelligence anxiety was measured using the Artificial Intelligence Anxiety Scale (AIAS), originally developed by Wang and Wang in 2019 to assess individuals’ anxiety related to the development and use of artificial intelligence technologies [18]. The scale comprises 21 items rated on a 7-point Likert scale (1 = never to 7 = completely) and evaluates four subdimensions—learning, job replacement, sociotechnical blindness, and AI configuration—within a single multidimensional structure that captures anxiety related to learning AI tools, concerns about AI replacing human labor, misconceptions about AI autonomy and malfunction, and fear of humanoid or human-like AI technologies [18]. Higher scores indicate higher levels of AI-related anxiety. The original scale demonstrated excellent internal consistency, with Cronbach’s alpha coefficients of 0.974 for learning, 0.917 for job replacement, 0.917 for sociotechnical blindness, 0.916 for AI configuration, and 0.96 for the total scale. The Turkish adaptation by Terzi (2020) further confirmed the validity and reliability of the instrument, preserving its four-factor structure and strong psychometric properties [13]. In the present study, the AIAS was used to quantitatively assess oncology nurses’ anxiety related to artificial intelligence and its potential impact on their professional roles.

#### 2.2.4. The General Attitudes Toward Artificial Intelligence Scale (GAAIS)

Attitudes toward artificial intelligence were measured using the General Attitudes toward Artificial Intelligence Scale (GAAIS), originally developed by Schepman and Rodway in 2020 to evaluate individuals’ overall perceptions of AI Technologies [19,20]. The scale includes 20 items rated on a five-point Likert scale (1 = strongly disagree to 5 = strongly agree) and consists of two subdimensions: Positive GAAIS (12 items), assessing favorable attitudes toward AI’s benefits and usefulness, and Negative GAAIS (8 items), capturing concerns, fears, and reservations regarding AI, with all negative items reverse-scored so that higher scores indicate more positive attitudes. The original scale demonstrated strong internal consistency (Positive GAAIS α = 0.88; Negative GAAIS α = 0.83). For the current study, the Turkish version of the GAAIS—adapted by Kaya et al. using a standardized translation and back-translation method—was utilized, and its psychometric adequacy was supported in the adaptation study [14]. In this research, the GAAIS was employed to assess oncology nurses’ attitudes toward artificial intelligence and to investigate how these attitudes relate to their AI readiness and AI-related anxiety. Although both instruments (MAIRS-MS and GAAIS) relate to perceptions of artificial intelligence, they measure conceptually distinct constructs. The MAIRS-MS assesses readiness for artificial intelligence, referring to individuals’ knowledge, perceived competence, and preparedness to engage with AI technologies in clinical practice, whereas the GAAIS evaluates attitudes toward artificial intelligence, reflecting individuals’ evaluative beliefs and emotional responses regarding the potential benefits and risks of AI technologies.

### 2.3. Statistical Analysis

Data were analyzed using the JAMOVI software (version 2.6.17). The Shapiro–Wilk test was employed to assess the normality of data distribution. Parametric tests were applied for variables that followed a normal distribution, whereas non-parametric tests were used for data that did not meet the assumption of normality. Spearman correlation analysis, the general linear model, and conditional mediation analysis were performed to examine the relationships among variables. A *p*-value of <0.05 was considered statistically significant.

## 3. Results

Table 1 presents the sociodemographic characteristics of the participants and their views on artificial intelligence. Of the 207 nurses included in the study, the majority were female (78.7%) and single (67.1%). Most participants held a bachelor’s degree (81.2%), and more than half had 1–5 years of experience in the oncology field (56.0%). A total of 69.1% of the nurses reported that they follow professional publications in their field. Only 12.1% of the participants stated that they had previously received training in artificial intelligence. Slightly more than half of the nurses (53.1%) reported an interest in using artificial intelligence, and 50.7% believed that artificial intelligence and robot nurses should be included in nursing education. While only 9.2% of the participants believed that artificial intelligence and robot nurses could replace nurses, more than half thought that artificial intelligence and/or robot nurses would be beneficial to the nursing profession (56.5%) and would reduce nurses’ workload (66.2%). Furthermore, 34.3% believed that artificial intelligence and/or robot nurses would increase patient satisfaction, and 42.0% thought that they would improve the quality of nursing care. Nearly half of the nurses (48.3%) reported concerns about future ethical issues related to the use of artificial intelligence in healthcare, and 30.4% stated that they would prefer to work with robot nurses.

**Figure 1:**
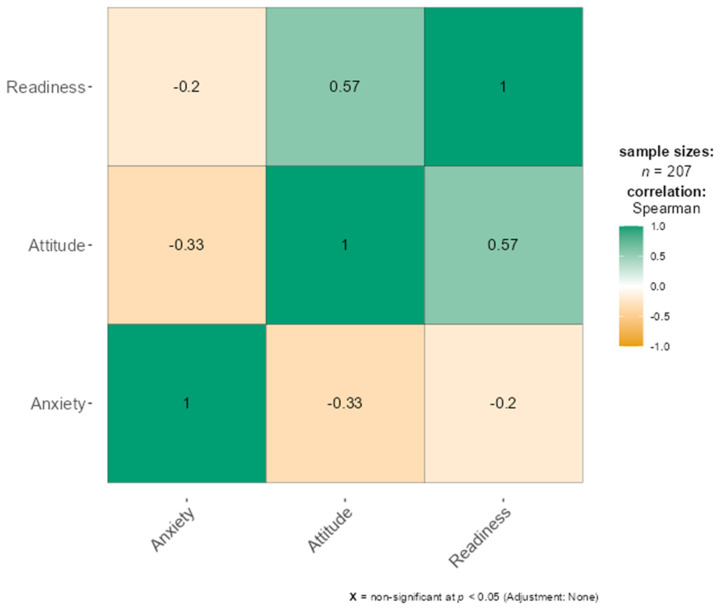
Correlations between anxiety, readiness and attitudes.

Table 2 presents the correlations between artificial intelligence anxiety (AIAS), readiness for artificial intelligence (MAIRS-MS), and attitudes toward artificial intelligence (GAAIS). Total anxiety was significantly and negatively correlated with skill (r = −0.240, *p* < 0.001), foresight (r = −0.155, *p* < 0.05), ethics (r = −0.204, *p* < 0.01), and total readiness scores (r = −0.188, *p* < 0.01). Learning-related anxiety showed moderate and significant negative correlations with skill (r = −0.337, *p* < 0.001), foresight (r = −0.248, *p* < 0.001), ethics (r = −0.269, *p* < 0.001), and total readiness (r = −0.246, *p* < 0.001). A strong and significant negative correlation was found between total anxiety and total attitude scores (r = −0.333, *p* < 0.001), with a particularly stronger association observed for positive attitudes (r = −0.390, *p* < 0.001). Similarly, significant negative correlations were identified between AI configuration anxiety, sociotechnical blindness anxiety, and attitude scores. Overall, the findings indicate that higher levels of readiness and more positive attitudes toward artificial intelligence are associated with lower levels of AI-related anxiety (Figure 1).

Table 3 presents the results of the general linear model examining the predictors of artificial intelligence anxiety (AIAS). Attitude toward artificial intelligence was found to be a significant predictor of anxiety (B = −0.939, SE = 0.228, 95% CI: −1.388 to −0.490, β = −0.327, t = −4.12, *p* < 0.001). This indicates that higher attitude scores were associated with significantly lower anxiety levels. In contrast, readiness for artificial intelligence was not a statistically significant predictor of anxiety (B = −0.187, SE = 0.138, 95% CI: −0.459 to 0.085, β = −0.107, t = −1.35, *p* = 0.178). These findings suggest that attitudes toward artificial intelligence play a stronger role than readiness in predicting anxiety.

Table 4 presents the estimated marginal means of total anxiety scores according to attitude levels. When other effects in the model were held constant at their mean values, the estimated mean anxiety score was 79.8 (SE = 2.92, 95% CI: 74.1–85.6) for individuals with low attitude levels (Mean − 1 SD). At the mean level of attitude, the estimated anxiety score was 70.5 (SE = 1.83, 95% CI: 66.8–74.1). For individuals with high attitude levels (Mean + 1 SD), the estimated mean anxiety score decreased to 61.1 (SE = 2.92, 95% CI: 55.3–66.8). These findings indicate a clear decreasing trend in anxiety levels as attitudes toward artificial intelligence become more positive.

**Figure 2:**
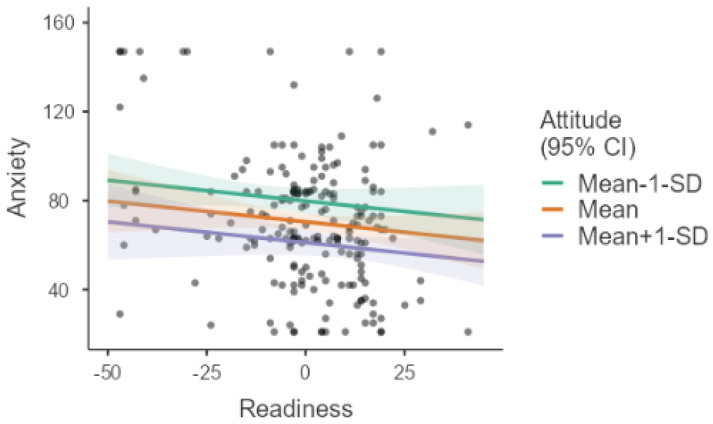
Moderating effect of attitude on the relationship between readiness and artificial intelligence anxiety.

Figure 2 illustrates the moderating effect of attitude on the relationship between readiness and artificial intelligence anxiety. The findings indicate that as positive attitudes increase, the negative association between readiness and anxiety becomes stronger. While the relationship between readiness and anxiety is relatively weak at low levels of attitude, higher levels of attitude are associated with a more pronounced decrease in anxiety as readiness increases. These results suggest that attitude plays a moderating role in the relationship between readiness and AI-related anxiety.

The mediating effect of attitudes toward artificial intelligence on the relationship between readiness and artificial intelligence anxiety, as well as the moderating effect of having received AI-related training, were examined using conditional mediation analysis (Table 5). The results indicated that the direct effect of readiness on anxiety was not statistically significant (β = 0.128, *p* = 0.329). Similarly, the moderating effect of AI training on this direct relationship was not significant. In contrast, attitudes toward artificial intelligence were found to be a significant full mediator in the relationship between readiness and anxiety. At the average level, the indirect effect of readiness on anxiety through attitudes was significant (β = −0.261, *p* < 0.001). Readiness had a positive effect on attitudes (β = 0.612, *p* < 0.001), whereas attitudes had a significant negative effect on anxiety (β = −0.427, *p* < 0.001). The indirect effect was stronger among participants who had received AI-related training (β = −0.338, *p* = 0.006). In this group, readiness had a stronger positive effect on attitudes (β = 0.642, *p* = 0.003), and attitudes had a stronger negative effect on anxiety (β = −0.526, *p* < 0.001). Although the indirect effect was also significant among participants without AI training (β = −0.181, *p* < 0.001), the magnitude of the effect was smaller compared with the trained group. Regarding total effects, the relationship between readiness and anxiety was significant only in the group without AI training (β = −0.306, *p* < 0.001), whereas the total effect was not significant in the group with AI training (*p* = 0.926). Overall, these findings demonstrate that attitudes toward artificial intelligence function as a full mediator in the relationship between readiness and anxiety, while AI training serves as a moderator that strengthens the indirect effect.

**Figure 3:**
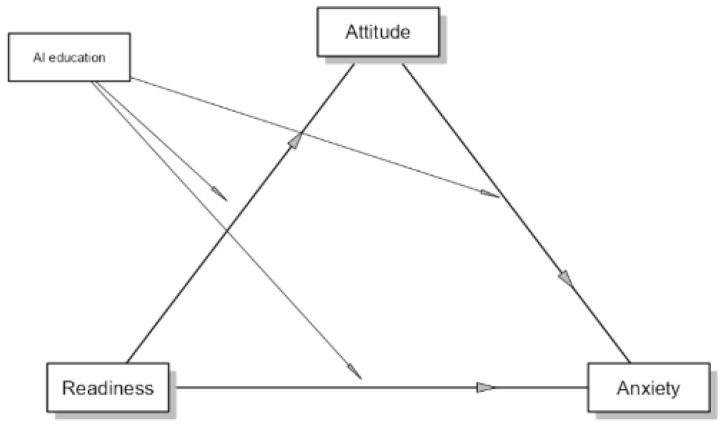
Conceptual diagram of the proposed mediation and moderation model.

Figure 3 illustrates the conceptual model of the conditional mediation analysis examining the mediating role of attitudes toward artificial intelligence in the relationship between readiness and artificial intelligence anxiety, as well as the moderating effect of AI-related education on this indirect pathway. In the model, readiness influences anxiety indirectly through attitudes, while AI education conditionally moderates the paths between readiness and attitudes and between attitudes and anxiety.

## 4. Discussion

This study examined the interrelationships among artificial intelligence readiness, attitudes toward artificial intelligence, and AI-related anxiety among oncology nurses, further testing the mediating role of attitudes and the moderating role of AI-related education. The findings provide strong empirical evidence that psychological constructs—particularly attitudes—appear to be associated with nurses’ emotional responses to artificial intelligence, beyond technical preparedness alone. This finding suggests that emotional responses to AI technologies may be shaped not only by technological competence but also by individuals’ cognitive evaluations and perceptions of technological change.

The descriptive findings indicate that although nurses largely perceive artificial intelligence as beneficial for reducing workload and improving care quality, they remain cautious regarding ethical implications and professional identity. This pattern is highly consistent with prior nursing studies reporting generally positive but ambivalent attitudes toward AI and robotics. For example, Booth et al. (2021) [21] reported that nurses tend to support AI-assisted care while expressing concerns related to accountability, patient safety, and ethical responsibility. Similarly, Topol (2019) [22] emphasized that healthcare professionals view AI primarily as a support tool rather than a replacement, aligning closely with the low proportion of nurses in this study who believed that AI could replace the nursing role. In the systematic review by El Arab et al. (2025) [23], nurses were reported to perceive artificial intelligence as a supportive tool capable of enhancing the quality of care; however, they also exhibited a cautious optimism toward this technology due to concerns related to ethical risks, data security, and the preservation of the professional nursing role. These findings confirm that nurses conceptualize AI as an assistive rather than substitutive technology, reinforcing the centrality of human judgment and caring in nursing practice. In the present study, this interpretation is supported by the descriptive results showing that only a small proportion of nurses believed that artificial intelligence could replace nurses, while a larger proportion reported that AI could reduce workload and support nursing practice. This pattern may reflect the tension between technological innovation and professional identity in nursing, where technological support is welcomed but concerns remain regarding the preservation of human-centered care.

The correlational findings showed that greater readiness and more positive attitudes toward artificial intelligence were consistently associated with lower levels of AI-related anxiety. A body of research has been conducted that has yielded findings analogous to those of the present study. Previous studies of this nature have established that positive attitudes towards artificial intelligence in nurses have been shown to engender a reduction in negative thoughts and concerns regarding the technology. In their 2025 study, Liu et al. [24] examined the relationship between AI literacy, attitudes, and AI anxiety among nurses. The findings of the study indicated that nurses with high AI literacy exhibited more positive attitudes and lower anxiety levels. Moreover, anxiety partially mediated the relationship between literacy and attitude [24]. In a cross-sectional study conducted by Al-Smadi et al. (2025), 400 children aged 13–16 in Jordan were examined [25]. The results demonstrated that the sub-dimensions of artificial intelligence anxiety significantly influenced attitudes towards artificial intelligence. The findings indicated that AI learning anxiety and AI structuring anxiety exhibited a positive correlation with positive attitudes, while job loss anxiety and socio-technical blindness demonstrated a significant negative impact on positive attitudes. It was also noted that negative attitudes could not be meaningfully explained directly by anxiety sub-dimensions, but were more likely related to emotional and cultural factors [25]. In a study conducted by Yiğit and Açıkgöz (2024), the researchers found that a lack of knowledge about artificial intelligence (AI), negative attitudes, and insufficient training significantly increased AI anxiety [26]. This finding was based on a study with nurse academics. Participants’ AI anxiety levels were found to be elevated; in particular, sociotechnical blindness, job loss, learning, and AI configuration anxiety subdimensions were found to be dominant. Furthermore, it was determined that AI anxiety increased with professional experience. One possible explanation for the observed increase in AI-related anxiety with greater professional experience may be related to concerns about changes in established clinical roles and professional autonomy. Experienced nurses may perceive emerging technologies as potentially altering long-standing clinical routines and decision-making authority, which may increase uncertainty regarding the future of professional practice. Previous studies have also suggested that healthcare professionals may experience anxiety about artificial intelligence due to concerns about job security and the impact of technological change on professional roles [27]. Furthermore, it was determined that anxiety was lower among individuals who received AI training. Yiğit et al. reported that nurses’ inadequate knowledge and skills regarding AI applications increased anxiety and negatively impacted AI acceptance [26]. A 2025 mixed-methods study of 500 nurses demonstrated that positive attitudes toward AI were significantly associated with lower resistance to change and anxiety, regardless of objective competency levels [28]. These findings lend further support to the hypothesis that a positive attitude and a state of readiness can serve as effective mechanisms in the reduction of anxiety, a phenomenon that was also observed in the present study. One possible explanation is that greater familiarity with AI reduces uncertainty and increases perceived control over technological systems, which may consequently decrease anxiety.

The findings of the present study demonstrate that attitudes toward artificial intelligence (AI) fully mediate the relationship between readiness and anxiety, while AI-related education functions as a significant moderator that strengthens this indirect effect. In line with this, a cross-sectional study conducted with 120 nurses reported that lower education levels and lack of familiarity with AI were associated with higher levels of AI anxiety, suggesting that both perceived competence and beliefs significantly influence anxiety [29]. Similarly, an ethics-focused educational intervention among nursing students was shown to significantly improve positive attitudes toward AI and intention to use AI, highlighting that education can reshape evaluative beliefs and thereby reduce anxiety [30]. A recent study among nursing students also reported generally positive attitudes toward AI and indicated that greater familiarity and education regarding AI were associated with reduced fear and greater acceptance of AI within nursing curricula [31]. Moreover, the moderating role of prior AI-related education observed in the present study is consistent with findings from a study conducted among family physicians, in which those with an information and communication technologies background or prior exposure to AI exhibited lower AI anxiety and higher readiness scores [32]. As in our study, educational background and training appear to shape how professionals internalize readiness as positive attitudes, which in turn influences anxiety and acceptance. This finding suggests that attitudes toward AI may function as a key psychological mechanism through which technological readiness translates into emotional responses to AI adoption.

## 5. Conclusions

Our findings suggest that while readiness for AI (competence, knowledge, perceived ability) is important, its association with AI anxiety appears to operate through attitudes toward AI. Attitudes—more than readiness itself—are central to whether nurses feel anxious about AI, and prior AI training strengthens this mechanism. Therefore, designing educational and implementation programs that foster positive and informed attitudes toward AI and address ethical concerns may help reduce anxiety and facilitate the safe integration of AI into nursing practice. The present study underscores that reducing AI-related anxiety requires not only technical competence but also structured educational programs that incorporate ethical dimensions. Structured education refers to training that includes AI literacy, technical competencies, and ethical considerations related to the use of artificial intelligence in healthcare. Sustainable educational models that strengthen nurses’ attitudes toward AI, enhance awareness, and alleviate anxiety are considered indispensable for the safe, ethical, and effective integration of AI into clinical practice. From a public health perspective, strengthening nurses’ AI readiness and attitudes may support the effective adoption of digital health technologies and contribute to improved healthcare service delivery.

### Strengths and Limitations

This study has several notable strengths. First, it employed well-validated measurement instruments (AIAS, MAIRS-MS, and GAAIS), which enhances the reliability and methodological rigor of the findings. Second, the inclusion of both mediation and moderation analyses allowed for a more nuanced examination of the psychological mechanisms linking readiness, attitudes, education, and AI-related anxiety. Third, focusing on practicing nurses in a highly relevant clinical field such as oncology strengthens the ecological validity of the results.

From a practical perspective, the findings provide important implications for nursing education and healthcare management. Developing educational programs that improve nurses’ AI literacy, technical competence, and ethical awareness may help reduce AI-related anxiety and facilitate the safe and effective integration of artificial intelligence into clinical practice. In addition, healthcare institutions may benefit from incorporating structured AI training into continuing professional development programs to support nurses’ preparedness for emerging digital health technologies.

Nevertheless, several limitations should be acknowledged. The cross-sectional design does not permit causal inferences; although the mediated model suggests a theoretically plausible directional pathway, longitudinal or interventional studies are required to confirm causality. In addition, the sample, while of adequate size, was drawn from a single institution or a limited number of hospitals, which may restrict the generalizability of the findings. The reliance on self-report measures also introduces the potential for social desirability bias, particularly as artificial intelligence becomes increasingly normalized in healthcare discourse. Finally, AI-related education was assessed using a dichotomous (yes/no) format; the frequency, depth, content, and quality of training were not evaluated, although these factors may substantially influence the magnitude of the observed moderating effect. In addition, participants were recruited through a WhatsApp group, which may introduce potential selection bias, as participation depended on voluntary responses and may have excluded nurses who were less active on digital communication platforms.

## Figures and Tables

**Table 1:** Distribution of the participants’ sociodemographic characteristics and their opinions on artificial intelligence.

Variable	n	%
**Gender**		
Female	163	78.7
Male	44	21.3
**Marital status**		
Married	68	32.9
Single	139	67.1
**Education level**		
Vocational Health High School	5	2.4
Associate degree	9	4.3
Bachelor’s degree	168	81.2
Master’s degree	25	12.1
**Length of work in oncology**		
Less than 1 year	72	34.8
1–5 years	116	56.0
More than 5 years	19	9.2
**Following professional publications**		
Yes	143	69.1
No	64	30.9
Previously received AI training	25	12.1
Interested in using AI	110	53.1
AI and robot nurses should be included in nursing education	105	50.7
AI and robot nurses can replace nurses	19	9.2
AI and/or robot nurses will be beneficial to the nursing profession	117	56.5
AI and/or robot nurses will reduce nurses’ workload	137	66.2
AI and/or robot nurses will increase patient satisfaction	71	34.3
AI and/or robot nurses will improve the quality of nursing care	87	42.0
Prefer using AI-based technologies	123	59.4
Prefer working with robot nurses	63	30.4
Concerned about future ethical issues related to AI in healthcare	100	48.3

**Table 2:** Correlations between anxiety (AIAS), readiness (MAIRS-MS), and attitudes (GAAIS) toward artificial intelligence.

Anxiety Dimensions (AIAS)	MAIRS-MS—Cognitive	MAIRS-MS—Skill	MAIRS-MS—Foresight	MAIRS-MS—Ethics	MAIRS-MS—Total	GAAIS—Positive Attitude	GAAIS—Negative Attitude	GAAIS—Total
**Learning**	−0.114	−0.337 ***	−0.216 **	−0.248 ***	−0.269 ***	−0.246 ***	−0.351 ***	−0.386 ***
Job Replacement	−0.126	−0.182 **	−0.149 *	−0.124	−0.157 *	−0.121	−0.351 ***	−0.256 ***
Sociotechnical Blindness Anxiety	−0.103	−0.102	−0.096	0.004	−0.090	−0.079	−0.240 ***	−0.169 *
AI Configuration	−0.137 *	−0.205 **	−0.172 *	−0.151 *	−0.191 **	−0.192 **	−0.360 ***	−0.327 ***
**Total Anxiety**	−0.130	−0.240 ***	−0.176 *	−0.155 *	−0.204 **	−0.188 **	−0.390 ***	−0.333 ***

AI = Artificial Intelligence. AIAS = Artificial Intelligence Anxiety Scale. GAAIS = General Attitudes toward Artificial Intelligence Scale. MAIRS-MS = Medical Artificial Intelligence Readiness Scale for Medical Students. Correlation test: Spearman correlation. Significance levels: * *p* < 0.05, ** *p* < 0.01, *** *p* < 0.001.

**Table 3:** Predictors of anxiety based on the general linear model.

Fixed Effects Parameter Estimates								
			95% Confidence Interval					
Names	Estimate	SE	Lower	Upper	β	df	t	*p*
(Intercept)	70.454	1.834	66.837	74.0711	0.000	204	38.41	<0.001
Attitude	−0.939	0.228	−1.388	−0.4897	−0.327	204	−4.12	<0.001
Readiness	−0.187	0.138	−0.459	0.0854	−0.107	204	−1.35	0.178

**Table 4:** Estimated marginal means.

Attitude					
				95% Confidence Interval	
Attitude (Moderator)	Mean	SE	df	Lower	Upper
Mean − 1-SD	79.8	2.92	204	74.1	85.6
Mean	70.5	1.83	204	66.8	74.1
Mean + 1-SD	61.1	2.92	204	55.3	66.8

Note. Means are estimated keeping other effects constant in the model.

**Table 5:** Conditional mediation analysis examining the mediating effect of attitudes and the moderating effect of AI training.

Moderator Levels					95% C.I.				
AI Education	Type	Effect	Estimate	SE	Lower	Upper	β	z	*p*
**Average**	**Indirect**	**Readiness** **⇒** **Attitude** **⇒** **Anxiety**	**−0.4699**	**0.1203**	**−0.706**	**−0.2342**	**−0.2614**	**−3.9071**	**<** **0** **.001**
Average	Component	Readiness ⇒ Attitude	0.3710	0.0685	0.237	0.5053	0.6120	5.4135	<0.001
Average		Attitude ⇒ Anxiety	−1.2667	0.2244	−1.706	−0.8268	−0.4272	−5.6446	<0.001
Average	Direct	Readiness ⇒ Anxiety	0.2308	0.2364	−0.233	0.6942	0.1284	0.9763	0.329
Average	Total	Readiness ⇒ Anxiety	−0.2460	0.2315	−0.700	0.2077	−0.1412	−1.0628	0.288
**Yes**	**Indirect**	**Readiness** **⇒** **Attitude** **⇒** **Anxiety**	**−0.6400**	**0.2339**	**−1.098**	**−0.1816**	**−0.3379**	**−2.7363**	**0.006**
**Yes**	Component	Readiness ⇒ Attitude	0.3893	0.1320	0.131	0.6480	0.6423	2.9498	0.003
**Yes**		Attitude ⇒ Anxiety	−1.6439	0.2244	−2.084	−1.2041	−0.5261	−7.3256	<0.001
**Yes**	Direct	Readiness ⇒ Anxiety	0.6813	0.4350	−0.171	1.5338	0.3597	1.5664	0.117
**Yes**	Total	Readiness ⇒ Anxiety	0.0413	0.4458	−0.832	0.9150	0.0237	0.0927	0.926
No	Indirect	Readiness ⇒ Attitude ⇒ Anxiety	−0.3136	0.0857	−0.482	−0.1457	−0.1808	−3.6604	<0.001
No	Component	Readiness ⇒ Attitude	0.3526	0.0370	0.280	0.4251	0.5818	9.5417	<0.001
No		Attitude ⇒ Anxiety	−0.8894	0.2244	−1.329	−0.4496	−0.3107	−3.9636	<0.001
No	Direct	Readiness ⇒ Anxiety	−0.2197	0.1432	−0.500	0.0609	−0.1266	−1.5343	0.125
No	Total	Readiness ⇒ Anxiety	−0.5333	0.1248	−0.778	−0.2887	−0.3062	−4.2725	<0.001

Note. Confidence intervals computed with method: Standard (Delta method). Betas are completely standardized effect sizes.

## Data Availability

The raw data supporting the conclusions of this article will be made available by the authors on request.
